# Preparation for Practice: Facilitating the Transition From Student to Physician Associate

**DOI:** 10.1111/tct.70176

**Published:** 2025-07-31

**Authors:** J. F. Lavallée, R. Horne, H. Scotchburn, K. Elliott, S. C. Shepherd

**Affiliations:** ^1^ Division of Medical Education, School of Medical Sciences University of Manchester Manchester UK

**Keywords:** allied health education, communication skills, curriculum evaluation

## Abstract

**Background:**

Physician Associate students find the transition from student to practitioner challenging, and universities need to adequately prepare students for clinical practice. We explored whether a simulation‐based course is an effective way of preparing final year Physician Associate students for practice as a qualified clinician, with a specific focus on developing their clinical communication skills.

**Approach:**

We piloted a new 1‐day ‘preparation for practice’ course for Physician Associate students nearing the end of their studies. The clinical communication skills team, academics on the Physician Associate course, guest tutors and simulated patients facilitated the course which followed one patient's healthcare journey over the span of a fortnight. The session included seven simulated events: handover, patient admission, requesting a scan, responding to an adverse event, talking with a dying patient, breaking bad news to the family, as well as explaining the process of the coroner and death certificate to a family member. The final aspect of the course was an open discussion with Physician Associates working in the NHS, involving a question‐and‐answer session, focusing on wellbeing.

**Evaluation:**

A total of 57 final year Physician Associate students attended, and we collected feedback from 56 students. All of the students rated the course content as highly useful, and 96% of students rated the quality of the course as high. Students reported increased confidence in complex clinical communication skills (*p* < 0.001).

**Implications:**

A preparation for practice course, including simulation and role modelling, is an effective way to practise complex clinical communication scenarios and improve students' confidence.

## 1 Background

A Physician Associate (PA) is defined as a healthcare professional who works as part of a multi‐disciplinary team under the supervision of a senior doctor, either a consultant or a general practitioner [1]. The role of the PA is well established in many countries such as the United States, where they are known as Physician Assistants. PAs were first introduced in the United Kingdom in 2003. PAs can provide care in both primary and secondary care settings, where their role includes taking medical histories, carrying out physical examinations and formulating differential diagnoses and management plans [1].

To become a PA, postgraduate study is required over 2 years and involves both academic study and clinical placements, where students are trained in a ‘medical model’. An undergraduate degree in a related subject (e.g., biomedical science, nursing) must be completed prior to commencing a PA course. Over the past 15 years, the number of PA training programmes has expanded rapidly [2].

Preparing all healthcare professional students for practice is vital, yet many graduate feeling unprepared, especially with more complex tasks, finding the transition challenging [3]. Developing a professional identity is fundamental during the transition from layperson to professional, enabling healthcare professionals to act with confidence [4].

Ensuring there are multiple opportunities to practise a variety of clinical encounters is valuable in healthcare training. Using simulation is an effective and enjoyable way to practise and build confidence and competence for healthcare professions [5]. Currently, there is little research exploring clinical communication skills training for PA students. We piloted a new ‘Preparation for Practice’ course which included simulated scenarios to enable students to experience challenges common within practice. We explored whether this 1‐day session could improve the students' confidence in complex clinical communication skills and better prepare them for clinical practice.

## 2 Approach

### 2.1 Course Design and Delivery

We designed and delivered a 1‐day ‘preparation for practice’ session for 57 final year PA students from a Northern UK University. The session was mandatory and held before their final exams to facilitate revision and the transition into professional roles. The session was carefully designed to simulate various challenging clinical situations that PAs may encounter post‐qualification but may not have had the chance to practise when on placement.

The session was modelled on the ‘preparation for practice’ session delivered to final year medical students, and the content informed by feedback received from former students stating the need for practical experience in a safe learning environment. The 1‐day session included discussion and simulation surrounding seven stations. During the simulations, students assumed the role of a qualified PA and followed one patient's journey from an outpatient scan to death, including handover, patient admission, requesting a scan, responding to adverse events, communicating with the dying patient, delivering bad news to the family and explaining the coroner's process and death certificate to a family member (Table 1).

**TABLE 1:** Station aims and content.

Station and aim	Station content
**(1) Handover** *Aim: To highlight the importance of clear communication, structure and preparation*.	Revision of Situation, Assessment, Background, Response (SBAR) approach Student activities: Organise information received in handover using SBAR format Prioritisation task for three patients Simulation: handover three patients Discussion: Importance of effective SBAR handover How to prioritise appropriately
**(2) Patient admission** *Aim: To negotiate admission with patient, taking into account deterioration is an indication for a change of plan and considering social care implications*.	Simulation: speak with patient to explain the change of plan and the need for admission Discussion: Clinical communication skills needed and used Support available for patients who are informal carers.
**(3) Requesting a scan** *Aim: To appreciate the importance of structured and clear communication and teamwork*.	Simulation: practise using SBAR when requesting scan Discussion: Importance of teamwork, prioritisation, communication, compassion and managing frustration.
**(4) Adverse event** *Aim: To identify and report medication errors* .	Simulation: to explore hypoglycaemic event with patient Discussion: Highlighting the importance of checking what medications the patient is taking and their concordance. Demonstrating the need for candour and considering next steps.
**(5) Talking with a dying patient** *Aim: To understand the role and importance of preparation before having difficult conversations* .	Simulation: to explore and respond to any concerns the patient has. Discussion: How to prepare for difficult conversations, including the environment How to answer questions from the patient How to debrief with a colleague afterwards
**(6) Breaking bad news** *Aim: To practise breaking bad news using the SPIKES model and honour the wishes of the patient*.	Simulation: to speak with relative, addressing their concerns and decide what information (if any) to disclose Discussion: What is confidentiality? How confidentiality is maintained when speaking with family members.
**(7) Death certificate and coroner (discussion with relative)** *Aim: To manage anger and resolve complaint*	Simulation: explaining the need to report the death to the medical examiner with the possibility of a referral to the coroner. Discussion: ensuring the students are aware of the processes involved and the roles of the medical examiner, bereavement centre and coroner.
**(8) Looking after your own health and wellbeing** *Aim: To discuss strategies to support health and wellbeing*	Discussions about what they are looking forward to once qualifying, signs of burnout and how to mitigate against this.
**(9) Question and answer session with professionals working in the NHS** *Aim: For students to ask questions relating to working clinically*	Opportunity for students to ask working Physician Associates about work life to demystify this experience and enable a smoother transition from student to worker.

We split the students into groups of approximately eight and each group was facilitated by a PA working in the NHS and clinical communication skills experts. Students were informed that the session was designed to be a safe space for them to trial different clinical communication skills and evaluate how they personally managed in these situations. Feedback was provided directly to the practising student at the end of each station, following Pendleton's Rules [6] and Agenda‐Led, Outcome‐Based Analysis (ALOBA; [7]). Within each station we included facilitated feedback discussions to encourage open and honest reflection, with support from experts, following a challenging discussion. We described this as a debrief session, with the aim of establishing a foundation for clinical debriefing throughout their career. We ended the session with a discussion about the students' own health and wellbeing, and a ‘question and answer’ session with PAs and doctors working in the NHS to facilitate safe and open discussion regarding student concerns.

## 3 Evaluation

### 3.1 Course Evaluation

We adapted the feedback form developed by Teagle et al. [8]. Completing the form was optional and anonymous. Question 1a was completed before the session, with all other questions completed after the session. We asked students to rate their confidence in complex clinical communication skills before and after the session using a 5‐point Likert scale, where 1 indicated not confident and 5 indicated very confident. Students rated the session using a Likert scale from 1 (*lowest*) to 5 (*highest*) in terms of course quality, content relevance and each scenario. We included two open‐ended questions asking the students to state what they enjoyed about the session and what could be improved. Quantitative data were analysed using descriptive statistics (e.g., means) and a paired samples *t*‐test to assess whether confidence changed significantly.

All students (*N* = 56) rated the session content as highly useful (a rating of 4 or 5), and 96% of students rated the quality of the session as high (a rating of 4 or 5). The students rated the quality and usefulness of each individual station highly (Table 2), with the ‘breaking bad news’ station rated highest, and the patient admission station rated lowest.

**TABLE 2:** Student ratings for the individual scenarios.

Simulated scenario	Student scoring^a^
(1) Handover	4.5
(2) Patient admission	4.4
(3) Requesting a scan	4.5
(4) Adverse event	4.5
(5) Talking with a dying patient	4.6
(6) Breaking bad news	4.7
(7) Death certifcate and coroner (discussion with relative)	4.5

^a^ 1 = low; 5 = high.

A paired samples *t*‐test (*α* = 0.05) was used to compare mean confidence ratings in handling complex clinical communication before *(M* = 2.8, SD = 0.58) and after (*M* = 4, SD = 0.5) completing the preparation for the practice session. On average, students self‐reported levels of confidence increased following the teaching session and this difference was statistically significant, *t*(55) = −16.142, *p* < 0.001, and medium, *d* = 0.6.

*Students self‐reported levels of confidence increased following the teaching session*.

As we only included two open ended questions, and due to the nature of the questions and research aims, we have presented the qualitative data verbatim rather than analysing as one cohesive dataset [9]. The qualitative feedback collected through free text comments focussed on the students' experience of practising challenging scenarios, including the pace of the session and receiving supportive feedback from PAs working in the NHS. Some example quotes are included in Table 3. The students stated that they would like more of these sessions, especially earlier in the academic year to enable them to practise these skills on clinical placement. They requested sessions that include more interprofessional communication, examination skills and medical documentation. A further suggestion was to include scenarios where there was a lack of understanding with regards to the role of a PA and developing skills to manage these situations.

**TABLE 3:** Qualitive feedback received in the free‐text boxes.

Feedback domain	Quotes from students
What the students liked about the session (*N* = 55)	I liked how there were different scenarios to work through and different communication skills to work on. The discussions we would have at each station were highly constructive and has vastly improved my confidence in handling complex comms. Great feedback, built my confidence. Tutor very supportive and helpful at making it realistic. Simulated patient practice. Well structured. Highlighted knowledge gap on death certificate/post‐mortems. Lots of different communication skills. Good teaching around SBAR, death certificates. Interesting having working PAs facilitate the session. It was great that the session was run by a practicing PA. Helpful insight into the reality of PA work.
Improvements the students would like ( *N* = 46)	Just to have more sessions for different things like skills, examinations More emphasis on medical documentation. Requesting scans etc. Have this session earlier and during multiple points during the year. Referrals, further practice on referring to other specialities. Quite like multiple of these through the year. Session about not wanting to be seen by a PA. Encountering scenarios where patient's do not trust PA role (explaining the role). Explaining procedures in conjunction to comms skills.

## 4 Implications

We developed a 1‐day ‘Preparation for practice’ session for PA students to facilitate their transition from university to the workplace using simulation, discussion and practical group work. A mixed methods systematic review of 30 studies demonstrated increased confidence, skills and knowledge in student education when simulation was used within healthcare training [10]. Providing a safe and controlled environment for students to develop and practise clinical communication is critical for simulated clinical communication skills training for PA students. Our session provided a safe environment for the students to gain confidence in handling more complex clinical situations and was evaluated as enjoyable, helpful and of being high quality. Providing a learning environment that fostered psychological safety was paramount to the success of this session. Having an open group discussion, known as a clinical debrief, after a challenging situation promoted reflection and helped learners understand and make sense of the event. Debriefings facilitated the learning of key lessons from simulated cases that can then be transferred to real life patient contexts. In addition, debriefings can reduce negative outcomes such as post‐traumatic stress symptoms [11] and burnout [12]. Therefore, effective debriefings, characterised by respect, trust and care for individuals, are paramount within student learning and future practice as a healthcare professional [13].

*Providing a learning environment that fostered psychological safety was paramount to the success of this session*.

The lack of understanding and negativity directed towards PA students during their training and career pathway from other members of staff can make the transition from student to qualified professional more difficult [14]. Identity formation amongst PA students is dynamic and complex but important [14]. Currently, many PA students and graduates are experiencing negativity regarding their role [15]. Our preparation for practice session highlighted the potential benefits of accessing PA role models and support during training to facilitate the group identity. Developing a strong professional identity is vital and can support students to respond effectively to challenges from staff and patients [14]. Students are seeking opportunities to develop their clinical communication skills in explaining their role within the multidisciplinary team and with patients, this will be an important addition to the clinical communication skills course in the future.

## Author Contributions

**Jacqueline F. Lavallée:** conceptualization, writing – original draft, formal analysis, data curation. **Rebecca Horne:** conceptualization, formal analysis, writing – review and editing. **Holly Scotchburn:** conceptualization, writing – review and editing. **Kirsty Elliott:** conceptualization, writing – review and editing. **Sarah C. Shepherd:** formal analysis, writing – review and editing, conceptualization.

## Ethics Statement

Approval by an ethics committee was not required as this was considered to be a teaching‐related evaluation where we did not collect any personal, sensitive or confidential information.

The University of Manchester accepts that low‐risk research or research‐like activities (e.g., evaluations) need a less rigorous approach to scrutiny and that a degree of proportionality should apply. Researchers must adhere to the published best practice guidelines concerning informed consent, data protection and mitigation of ethical issues.

## Conflicts of Interest

The authors declare no conflicts of interest.

## Data Availability

The data that support the findings of this study are available from the corresponding author upon reasonable request.
